# Targeting adipose tissue

**DOI:** 10.1186/1758-5996-4-43

**Published:** 2012-10-27

**Authors:** Bodo Haas, Paul Schlinkert, Peter Mayer, Niels Eckstein

**Affiliations:** 1 Federal Institute for Drugs and Medical Devices, Kurt-Georg-Kiesinger-Allee 3, Bonn, 53175, Germany; 2 University of Salzburg, Molecular Biology, Hellbrunnerstraße 34, Salzburg, 5020, Austria

**Keywords:** ‘Brite’ adipocytes, Brown adipose tissue, Positron emission tomography, Thermogenesis, Transdifferentiation, UCP1, White adipose tissue

## Abstract

Two different types of adipose tissues can be found in humans enabling them to respond to starvation and cold: white adipose tissue (WAT) is generally known and stores excess energy in the form of triacylglycerol (TG), insulates against cold, and serves as a mechanical cushion. Brown adipose tissue (BAT) helps newborns to cope with cold. BAT has the capacity to uncouple the mitochondrial respiratory chain, thereby generating heat rather than adenosine triphosphate (ATP). The previously widely held view was that BAT disappears rapidly after birth and is no longer present in adult humans. Using positron emission tomography (PET), however, it was recently shown that metabolically active BAT occurs in defined regions and scattered in WAT of the adult and possibly has an influence on whole-body energy homeostasis. In obese individuals adipose tissue is at the center of metabolic syndrome. Targeting of WAT by thiazolidinediones (TZDs), activators of peroxisome proliferator-activated receptor γ (PPARγ) a ‘master’ regulator of fat cell biology, is a current therapy for the treatment of type 2 diabetes. Since its unique capacity to increase energy consumption of the body and to dissipate surplus energy as heat, BAT offers new perspectives as a therapeutic target for the treatment of obesity and associated diseases such as type 2 diabetes and metabolic syndrome. Recent discoveries of new signaling pathways of BAT development give rise to new therapeutic possibilities in order to influence BAT content and activity.

## White adipose tissue as drug target

WAT is the largest lipid and energy storage in the human body. Excessive body fat, however, leads to insulin resistance, dyslipidaemia and type 2 diabetes. Over a long time it was believed that WAT is hormonally inert, but this paradigm is now obsolete. Today it is estimated, that the adipose organ is the largest endocrine organ of the human body [1]. Adipose tissue mainly produces and secretes cytokines such as adiponectin (encoded by the murine gene *Adipoq*), leptin (encoded by the murine gene *Lep*), resistin, tumor necrosis factor α (TNFα) and interleukins (ILs), which are referred to as adipokines according to their site of secretion [2]. In healthy (lean) subjects, adiponectin has a positive insulin sensitizing effect on other tissues [3]. Leptin is secreted by adipocytes and suppresses appetite by binding to leptin receptors in the central nervous system (CNS) [4]. In obesity, however, different adipokines take over control. Among others, resistin, IL-1, IL-6, and TNFα are secreted from adipocytes, fibroblasts, macrophages and monocytes which reside in adipose tissue. These adipokines are described to be involved in mediating insulin resistance in peripheral tissues and to increase the risk for type 2 diabetes [5-8]. Excess adiposity is not only associated with a dysregulation in adipokine profile, but also with an increased portal release of free fatty acids (FFA). The high concentrations of FFA decrease the hepatic degradation of apolipoprotein B and insulin, which may contribute to the dyslipidemia, hyperinsulinemia, and insulin resistance observed in visceral obesity [9].

**Figure 1:**
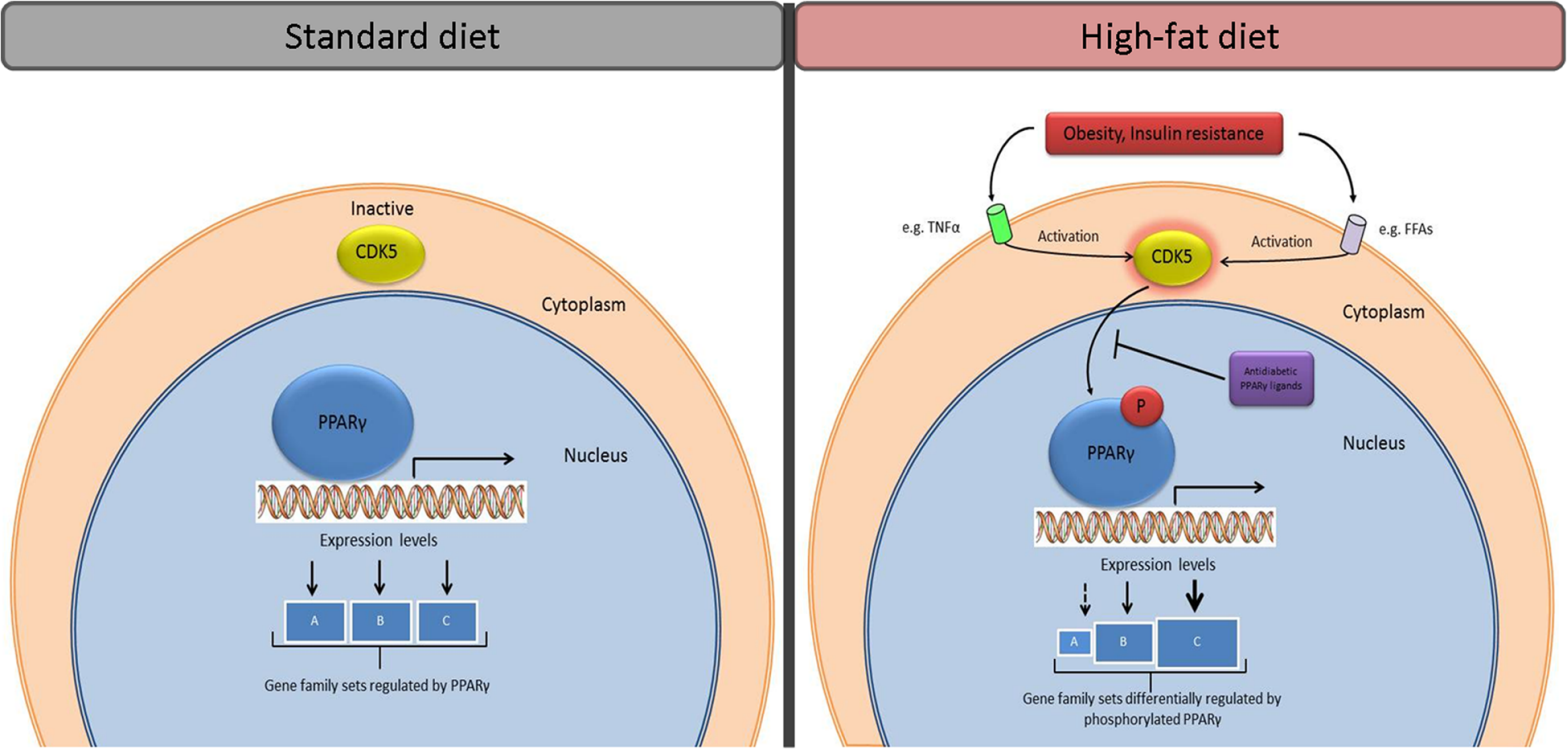
**Regulation of PPARγ activity in conditions of a standard diet (lean, insulin-sensitive state) compared to a high-fat diet (obese, insulin-resistant state) in WAT of mice.** The activity of PPARγ is differentially regulated during standard diet (left) and high-fat diet (right) conditions. Obesity and insulin resistance, both a result of a high-fat diet, lead to the production of inflammatory cytokines, such as TNFα. Both FFA and TNFα can activate CDK5, which in turn phosphorylates serine 273 on PPARγ thereby inducing the expression of different gene family sets compared to unphosphorylated PPARγ. Differential induction and repression of gene sets are represented by A, B, and C. Furthermore, some PPARγ ligands block the phosphorylation of PPARγ by CDK5, which may result in the expression of the same gene family as under a normal diet. Abbreviations: CDK5, cyclin-dependent kinase 5; FFA, free fatty acids; PPARγ, peroxisome proliferator-activated receptor γ; TNFα, tumor necrosis factor α.

A current pharmacological approach to counteract insulin resistance and type 2 diabetes is the use of thiazolidinediones (TZDs), which are agonists on peroxisome proliferator-activated receptor γ (PPARγ). TZDs promote differentiation of preadipocytes into white adipocytes [10] and are assumed to redirect FFA away from skeletal muscle towards adipose tissue (the so-called lipid steal hypothesis) [11,12]. TZDs are also implicated in positively influencing insulin resistance by decreasing the expression of TNFα, IL-1, and resistin while increasing the production of adiponectin [2,13]. A drawback of this therapeutic approach is weight gain caused by the accumulation of differentiated adipocytes, fluid retention, and an increased risk of cardiovascular events. These side effects are probably caused by the classical agonist action of TZDs. Choi et al. [14] have currently described a differential regulation of PPARγ. Ligands which prevent the high-fat diet (TNFα)-induced phosphorylation of Ser 273 of PPARγ by CDK5 and only have minimal agonist activity still retain a potent anti-diabetic effect [14,15]. Phosphorylation of PPARγ by CDK5 does not change its transcriptional activity *per se* but changes the expression of specific genes (e.g. reduction in *Adipoq* expression) (Figure 1).

Promising partial PPARγ agonists such as NS-1 (5Z)-5-[4-hydroxy-3-methoxy-phenyl) methylene] thiazolidine-2, 4-dione) or balaglitazone are currently investigated [16,17]. NS-1 induced adipogenesis *in vitro* to a 25 fold lesser extent than pioglitazone and improved hyperglycemia and insulin resistance in diet-induced obesity mice without significant weight gain. Balaglitazone already underwent phase III clinical trials and showed significant improvement in glucose handling and hemoglobin A1c (HbA1c) levels comparable to pioglitazone. Importantly, side effects like fluid retention and fat accumulation were less pronounced in the balaglitazone groups [18]. Thus, identification of ‘partial’ PPARγ agonists which prevent phosphorylation of Ser 273 but do not show classical agonist action might lead to more specific therapies reducing the emergence of side effects [14].

Another class of antidiabetic drugs, the sulfonylureas, which stimulate insulin secretion by blocking ATP-dependent potassium channels of pancreatic β-cells, have also been implicated to act on human white adipocytes. It has been demonstrated that glimeperide and glibencalmide induce the differentiation of human white preadipocytes and suppress cytokine expression probably by activation of PPARγ [19].

Another approach to target adipocytes is to increase lipolysis. Lipolysis in adipocytes is mediated by norepinephrin (NE) [20]. NE binds to β-adrenoceptors (β-ARs), which are highly abundant on the surface of adipocytes, and thereby activates adenylate cyclase (AC) which increases intracellular cAMP levels. As a consequence hormone-sensitive lipase (HSL) and perilipin are phosphorylated by PKA leading to the release of FFA. However, FFA themselves could increase insulin resistance, as mentioned above. This process is tightly regulated by the cGMP-inhibited phosphodiesterase 3B (PDE3B), which is expressed in white and brown adipocytes. Activation of PDE3B leads to increased hydrolysis of cAMP and thereby inhibition of catecholamine-induced lipolysis, reduction in insulin-induced glucose uptake, and lipogenesis (reviewed in [21]). Using the PDE3 inhibitor amrinone it could be shown that insulin's antilipolytic effect on human adipose tissue *in vivo* is mediated by stimulation of PDE3 [22]. Targeted-inhibition of PDE3B in conjunction with a β-AR agonist could therefore be a promising approach to increase intracellular cAMP levels in adipocytes. However, to date it is not known whether this approach is feasible due to the release of FFA. Furthermore, β-AR agonists and PDE inhibitors could have cardiovascular side effects.

### Brown adipose tissue physiology

In contrast to WAT, brown adipose tissue (BAT) combusts energy and releases heat. This unique function allows newborns to maintain body temperature in cold environments (non-shivering thermogenesis). BAT and WAT differ not only at the functional but also on the morphological and molecular levels. White adipocytes are usually large, round cells with a diameter that varies from 25–200 micrometer, and contain a large unilocular lipid droplet surrounded by a thin layer of cytoplasm with few mitochondria. The nucleus is flattened and located on the periphery. In contrast, brown adipocytes are cells with polygonal shape, normal cytoplasm with multilocular lipid droplets, and the nucleus is located centrally. Their brown color is due to the large quantity of mitochondria exhibiting numerous cristae. The main biological function of brown adipocytes is thermogenesis; energy storage in the form of lipids takes place but to a lesser extent to that of white adipocytes [23].

In infants, BAT is mainly localized in the neck, supraclavicular, in the axillae, and peri-/suprarenal. After birth BAT content is high accounting for 2–5% of body weight but gradually decreases during the first years [24,25].

The ability to generate heat (thermogenic capacity) results from a protein referred to as uncoupling protein 1 (UCP1) [26]. It is found exclusively in brown adipocytes, where it ensures uncoupling of the mitochondrial respiratory chain. UCP1 forms a pore in the inner mitochondrial membrane, leading to the break-down of the electrochemical proton gradient (proton leak), which is required for the activation of ATP synthesis in the mitochondrial matrix. As a result, ATP synthesis is blunt and excess energy is released as heat. UCP1 is activated among others by FFA and thus its activity is directly coupled to lipolysis. Cold stimuli lead to activation of the sympathetic nervous system (SNS) and the release of NE at the sympathetic nerve terminals, which profusely innervate the BAT. NE binds to β_3_-ARs, which are highly abundant on the surface of brown adipocytes, and activates lipolysis. The subsequent release of FFA activates UCP1. Additionally, NE increases mitochondrial biogenesis and the expression of *Ucp1* via activation of p38 mitogen-activated protein kinase (p38MAPK) and PPARγ coactivator 1α (PGC1α) [27]. Thus, prolonged cold exposure triggers brown adipocytes to produce more heat. Brown adipocytes can sufficiently provide themselves with lipids as they express insulin receptors and glucose transporters and thus are able to take up larger amounts of glucose, a substrate for lipogenesis. The insulin pathway is crucial for the regulation of adipogenesis, *Ucp1* expression, and mitochondrial biogenesis [28]. Insulin can further affect thermogenesis by increasing substrate uptake by BAT and increasing sympathetic activity mediated by the hypothalamus [29]. These factors may be connected to the thermic effect of food [30]. In experiments with brown adipocytes derived from mice it was demonstrated that nitric oxide (NO) and cyclic guanosine monophosphate (cGMP) can increase *Ucp1* expression and mitochondrial biogenesis via protein kinase G (PKG). Simultaneously, the activated cGMP pathway increases the differentiation of preadipocytes into mature brown adipocytes by interfering with the RhoA/ROCK and insulin signaling pathways [31,32]. Also regulation of the cGMP cascade in BAT and WAT has recently been shown for natriuretic peptides (NPs) via the NP receptor A (NPRA) and the NP clearance receptor (NPRC) [33]. In contrast, Becerril et al. [34] demonstrated, that inducible NO synthase (*iNOS*)-knockout mice showed an increased expression of genes involved in brown adipocyte function (*Pgc1a, Ucp1, and Ucp3*) and brown adipogenesis (*Prdm16* and *Bmp7*). Moreover, deletion of the *iNOS* gene improves the brown-like phenotype and the molecular function of brown fat in *Lep*-deficient obese (*ob/ob*) mice, which is characterized by a “white-like” appearance of BAT. Thus, further studies are needed to disentangle the role of NO in the physiology of BAT.

**Figure 2:**
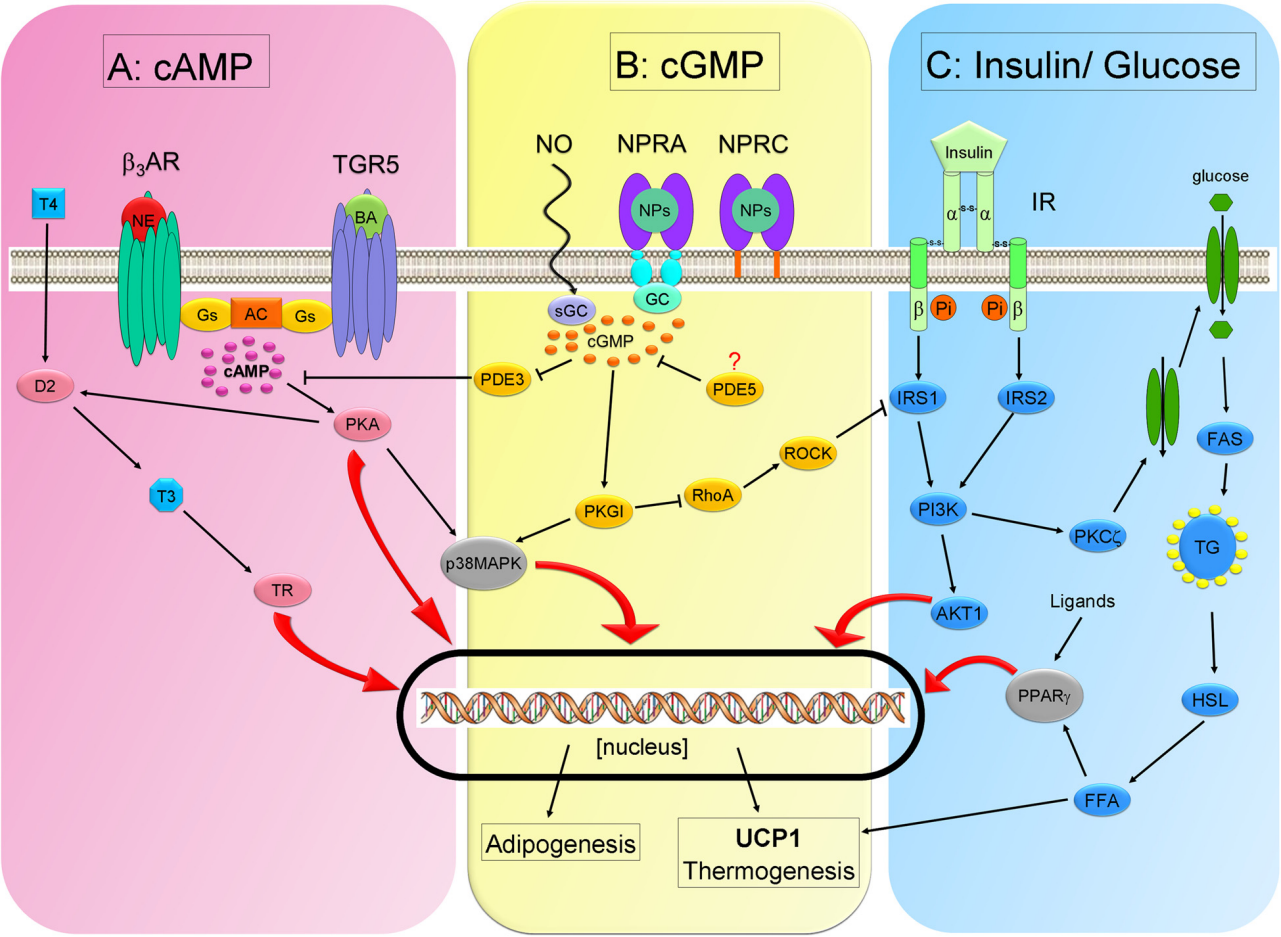
**Mechanisms of BAT activation.** Schematic overview of the complex signaling network leading to activation of BAT by exogenous stimuli. Abbreviations: AC, adenylate cyclase; AKT1, protein kinase B; BA, bile acids; cAMP, cyclic adenosine monophosphate; cGMP, cyclic guanosine monophosphate, D2, deiodinase type 2; FAS, fatty acid synthase; FFA, free fatty acids; GC, guanylate cyclase; Gs, stimulatory G protein; HSL, hormone-sensitive lipase; IR, insulin receptor; IRS, insulin receptor substrate NE, norepinephrin; NO, nitric oxide; NPs, natriuretic peptides; NPRA, NP repector A; NPRC, NP clearance receptor; p38MAPK, p38 mitogen-activated protein kinase; PDE, phosphodiesterase; PI3K, phosphoinositide 3 kinase; PKA, protein kinase A; PKCζ, protein kinase C ζ; PKGI, protein kinase G 1; RhoA, ras homolog gene family member A; ROCK, Rho-associated protein kinase; T4, thyroxine; T3, triiodothyronine; TG, triacylglycerol; TGR5, G-protein-coupled receptor; TR, thyroid hormone receptor; UCP1, uncoupling protein 1; β_3_AR, β3-adrenoceptor.

Brown adipocyte function is further regulated by thyroid hormones because they express high amounts of thyroid hormone receptors and the enzyme deiodinase type 2 (D2, encoded by the murine gene *Dio2*), which converts thyroxine (T4) into the more potent triiodothyronine (T3). T3 promotes mitochondrial biogenesis, induces the expression of *Ucp1,* and increases the activity of brown adipocytes [35,36]. Expression and activity of D2 can be triggered by cAMP. Bile acids can mediate cAMP-dependent D2 activation via the G-protein-coupled receptor TGR5 in brown adipocytes leading to an increase in energy expenditure [37] (Figure 2).

Thermogenesis and BAT activity is under tight control of the CNS. BAT is innervated by distinct nuclei of the hypothalamus, which coordinates signals from temperature changes and food intake [38]. A crucial role in hypothalamic regulation of sympathetic outflow and BAT thermogenesis plays AMP-activated protein kinase (AMPK). The inhibition of AMPK by leptin in the hypothalamus is an important mechanism in the regulation of food intake and energy homeostasis. Protein tyrosin phosphatase 1B (PTP1B) has been implicated in interfering with leptin signaling in the hypothalamus [39]. Using neuronal *Ptb1b* knock-out mice, Xue et al. [40] demonstrated that absence of *Ptb1b* in the hypothalamus results in increased leptin signaling and augmented inhibition of hypothalamic AMPK. As a consequence neuronal signaling to BAT is altered leading to increased AMPK activity in BAT with increased uncoupling and mitochondrial biogenesis. Inhibition of AMPK by central administration of T3 to rats leads to reduced body weight without altering food intake due to increased SNS activity and upregulation of thermogenic markers in BAT. Importantly, administration of T3 to a single nucleus of the hypothalamus could entirely replicate effects on BAT achieved with peripherally applied T3 in higher doses [41]. Currently, bone morphogenic protein 8B (BMP8B) has, besides its effect on brown adipocytes, also been described to have central effects on BAT. *Bmp8b*^−/−^ mice display altered neuropeptide levels and reduced phosphorylation of hypothalamic AMPK. Central BMP8B treatment resulted in increased thermogenesis in BAT via neuronal activation of regulatory nuclei in the hypothalamus [42].

### BAT in adult humans

In the last decade it could be demonstrated by using positron emission tomography (PET) that adult humans also possess metabolically active BAT [43-46]. Originally, PET was used in conjunction with computer tomography (CT) to image tumors and metastases [47]. Tumor tissues have a higher metabolic activity and thus take up more glucose, a phenomenon which is referred to as the Warburg effect. Cancer patients injected with radioactively labeled glucose (2-deoxy-2^18^F]fluoro-D-glucose, FDG), take up FDG in tumor tissue by sodium-independent glucose transporters (e.g. GLUT1, GLUT3 and GLUT4). After passing the initial phase of glucose metabolism and phosphorylation by hexokinase to the 6-phosphate form, FDG does not enter the Krebs cycle and is trapped in the cell. This results in a strong signal of tumor tissues in the PETscan. It was noted that in some PET scans other regions also showed a symmetrical, high FDG uptake, especially around the collar bone (supraclavicular), the mediastinum (para-aortic), along the spinal column (paravertebral), and in the kidneys (supra-/perirenal) [47,48]. After the combination of PET with CT (PET-CT), it soon became clear that the unusual pattern is not caused by muscles, but rather by a tissue with a lower density such as adipose tissue. White fat depots, however, show only marginal glucose uptake and hardly give a signal in PET-CT. It took a rather long time until it was concluded that the observed signals are BAT [48]. Additionally, subjects who were exposed to cold prior to PET scans, show an even stronger FDG uptake in BAT [45,49]. However, β-AR antagonists such as propranolol (~1 mg/kg) reduce FDG uptake [50,51]. Studies of biopsies from the supraclavicular region in 2009 finally provided clear proof that the excised tissue is BAT [46,52]. The expression of specific genes such as *UCP1* was shown first, followed by the histological examination, which showed the presence of multilocular lipid droplets, an unequivocal characteristic of BAT.

### Lineage development of white and brown adipocytes

**Figure 3:**
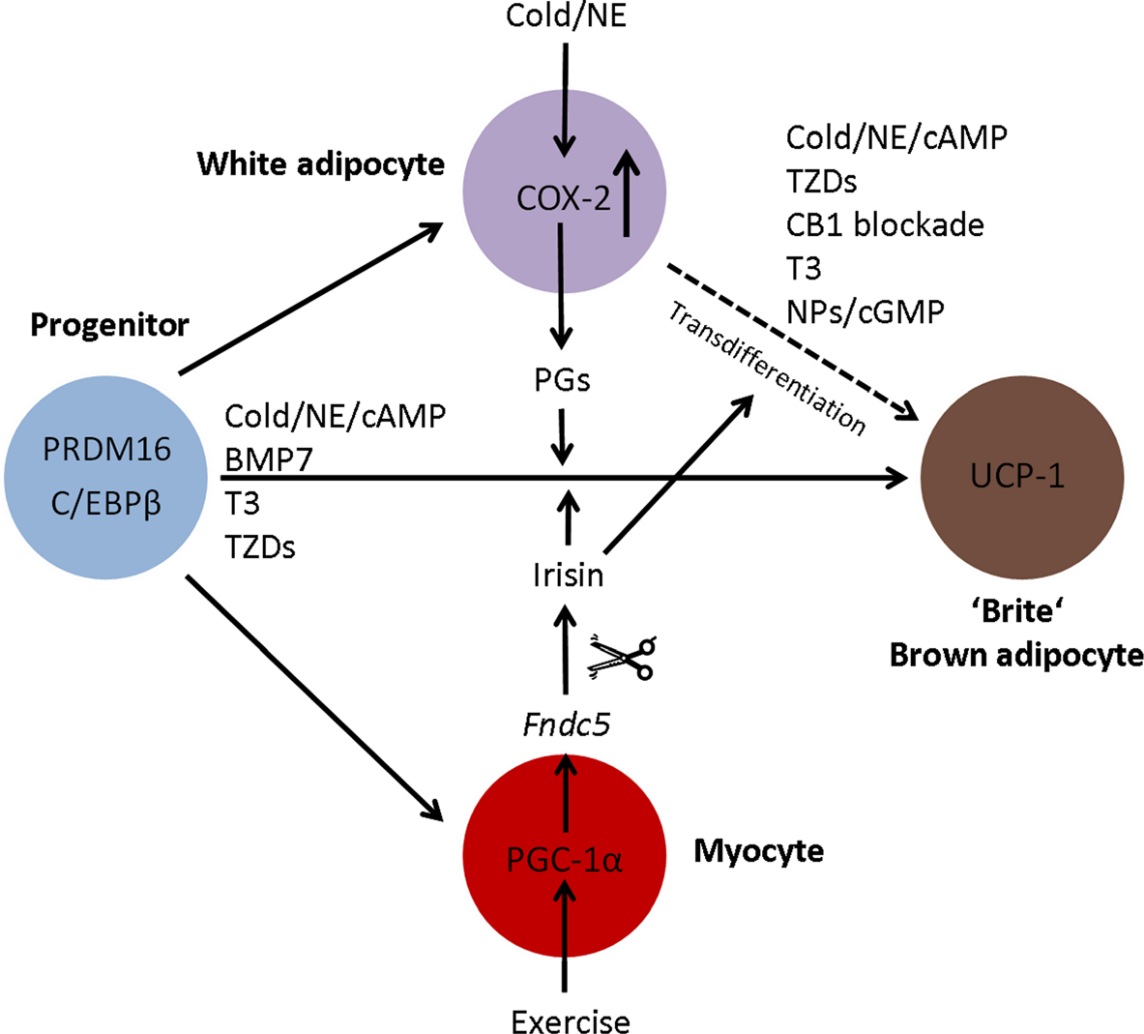
**Control of ‘brite’ (brown) adipocyte development.** The scheme depicts the interplay of white adipocytes, myocytes, and mesenchymal precursor cells involved in the (trans-) differentiation process of ‘brite’ and/or brown adipocytes. Abbreviations: BMP7, bone morphogenic protein 7; cAMP, cyclic adenosine monophosphate; CB1, cannabinoid type 1 receptor; C/EBPβ, CCAAT/enhancer binding protein β; cGMP, cyclic guanosine monophosphate; COX2, cyclooxygenase 2; *Fndc5*, fibronectin type III domain containing 5; NE, norepinephrin; NPs, natriuretic peptides; PGs, prostaglandines; PGC1α, PPARγ-coactivator 1α; PRDM16, PR domain containing 16; T3, triiodothyronine; TZDs, thiazolidinediones; UCP1, uncoupling protein 1.

Adipocytes derive from multipotent mesenchymal stem cells (MSCs), which emerge from the mesoderm of the embryo. White adipocytes differentiate from pericytes, which derive from the lateral mesoderm [53]. In the course of development adipoblasts (precursor cells) are derived from the MSCs which further develop into preadipocytes. Preadipocytes finally are able to differentiate into mature white adipocytes. A similar course was proposed for the development of brown adipocytes. Surprisingly, it has recently been found that brown adipocytes carry the genetic signature of a muscle cell. Muscle precursor cells derive from the paraxial mesoderm and express the gene *Myf5*. They can develop into both skeletal muscle cells and brown adipocytes [54,55]. A driving factor inducing the differentiation of progenitors (*Myf5-* positiv and -negativ) into brown adipocytes via the activation of transcription factors such as PR domain containing 16 (PRDM16), CCAAT/enhancer-binding protein β (C/EBPβ), and PGC1α is the cytokine BMP7, which belongs to the TGFβ superfamily [56,57]. It has been shown in lineage tracing studies that the interscapular and perirenal BAT depots of mice are derived from *Myf5*-positive muscle precursor cells. However, brown adipocytes can also appear in WAT and muscle tissue without carrying the *Myf5* signature, for example, after chronic cold exposure or β-adrenergic stimulation [58]. Both, transdifferentiation of white adipocytes into brown adipocytes (‘brown in white’ so called ‘brite’ or ‘beige’ adipocytes) and the occurrence of specific precursors of brown adipocytes in WAT are currently discussed (Figure 3). In a very recent publication by Wu et al. [59] it was demonstrated that ‘brite’ adipocytes have extremely low basal expression of *Ucp1*, but, like classical brown adipocytes, they respond to cAMP stimulation with high *Ucp1* expression and respiration rates. Data from *UCP1* expression studies suggest that a lean adult possesses one brown adipocyte scattered in 100–200 white adipocytes of visceral adipose tissue [60]. In a recent publication by Vegiopoulus et al. [61] cyclooxygenase 2 (COX2) activity and prostaglandines (PGs) have been implicated as downstream effectors of β-adrenergic signaling in WAT, which shift progenitor cells towards a brown adipocyte phenotype. Boström et al. [62] have shown that PGC-1α stimulated the expression of several muscle gene products such as the newly identified type I membrane protein irisin (encoded by the murine gene *Fndc5*), which was found in mouse and human plasma and is secreted after exercise. Overexpession of *Fndc5* in mice resulted in 3–4 fold increase of irisin plasma levels and induced browning of subcutaneous fat and thermogenesis. Moderately increased irisin expression in high-fat diet-treated obese and insulin-resistant mice resulted in reduction of body weight and improvement of diet-induced insulin resistance. Moreover, ‘brite’ adipocytes have been shown to be preferentially sensitive to irisin [59].

In another study by Ohno et al. [63] it has been shown that PPARγ agonists like rosiglitazone induce the browning of subcutaneous white adipose tissue through stabilization of the PRDM16 protein. NPs through NPRA, cGMP, and PKG can activate p38α MAPK to increase mitochondrial biogenesis and uncouple respiration in human white adipocytes. It has also been demonstrated that atrial NP and β-AR agonists can act together in an additive manner to more robustly promote brown adipocyte features and functions in human white adipocytes [33]. In this context it is noteworthy that expression of *Pde5* has been described in white adipocytes [64,65]. Inhibition of PDE5 with sildenafil triggered the differentiation of white preadipocytes [66]. Therefore, PDE5 blockade in adipose tissue should lead to increasing intracellular cGMP levels and could also have an impact on brown adipocyte differentiation and browning of WAT. This hypothesis is further strengthened by the finding that PDE5 inhibition with vardenafil in human WAT explants resulted in a marked increase in *PPARG* and *PGC1A* expression paired with an increase in mitochondrial DNA content [67]. However, data on *Pde5* expression in brown adipocytes are still lacking (reviewed in [68]). Blockade of cannabinoid type 1 receptor (CB1) with rimonabant, a selective antagonist of the CB1 receptor expressed on white adipocytes, induces transdifferentiation of differentiated murine white adipocytes towards a brown fat phenotype [69]. In addition to its function on BAT, T3 has also been implicated in the induction of brown-like adipocytes in WAT [70] (Figure 3).

### Physiological relevance of BAT in adults

The emerging question however is whether BAT in adults plays an essential physiological role. A retrospective analysis of studies with PET and PET-CT scans showed that under normal conditions BAT was only detected in about 10% of the examined adults [43,48]. However, histological analysis of biopsies revealed an approximately three times higher abundance [52]. Prospective studies, where young people were exposed to cold, concluded that 96% of the subjects showed functional BAT [45]. The controversy of these results is probably related to the inaccuracy of the PET-CT imaging technology. In addition, various factors such as ambient temperature, food intake or intake of FFA and the use of β-AR antagonists [50,51] can affect FDG uptake of the cells, which was not taken into account in any of the studies. The fact that BAT content decreases with age while usually WAT content increases with age, suggests, that BAT may play a role in whole-body energy homeostasis. In most studies, an inverse correlation of body mass index (BMI) and incidence of BAT and its activity were detected. From a bioenergetic perspective when activated (e.g. by cold exposure) BAT takes up about as much glucose as the brain, which is beside muscles, the main glucose consumer of the body. In an older review from the 1980s Stock and Rothwell [71] already calculated that “as little as 80–100 g of brown fat, producing heat at half its maximal capacity, could account for approximately 20% of a 70 kg man’s daily energy expenditure. Thus, this almost insignificant mass of tissue could make all the difference between him maintaining weight or gainig up to 20 kg per year.” More recent studies propose more moderate assumptions: In a PET-CT scan of a subject 63 g of supraclavicular BAT were detected. The authors concluded that if this deposit was fully activated over a year, it would burn energy equivalent to the energy content of about 4 kg of adipose tissue and thus would contribute substantially to energy expenditure [46]. Using a new approach by also measuring nonesterified fatty acid (NEFA) uptake upon cold exposure it could be shown that all subjects displayed substantial glucose and NEFA uptake in BAT [72]. An increase of 80% total energy expenditure was observed during 3 hours of cold exposure and it is likely that a big portion was mediated by BAT thermogenesis. The authors calculated that mobilization of 28 g of BAT lipid reserve would be sufficient to account for the extra energy of roundabout 250 kcal expended. Taken together, these studies demonstrate that BAT is undoubtedly involved in non-shivering thermogenesis in adults. As main sources of energy for this process BAT consumes intracellular TG and glucose. However, it remains to be determined whether frequent and chronic cold exposure leads to relevant energy expenditure or if compensatory mechanisms such as increased appetite might countervail this effect.

### Activation of BAT development and activity by drugs

Improvement of the imaging technology is essential to further identify (scattered) depots of BAT and measure its impact on energy expenditure for the treatment of obesity. A new approach has been reported by Chen et al. [73], which demonstrated the feasibility of measuring BAT volume and function *in vivo* by using magnetic resonance imaging in rats. Nevertheless, it is not clear yet whether human adults have sufficient BAT (or its precursor cells), which could be activated to have a significant long lasting effect on energy expenditure. Despite all uncertainties BAT appears to be a promising pharmacological target for the treatment of obesity and associated diseases like type 2 diabetes. Experiments in rats and mice give cause for hope that in humans the activation of specific signaling pathways in BAT can increase its development and activity and thus accelerate energy expenditure (extensively reviewed in [74]). Several approaches are possible (see also Table 1 for drug substances currently under investigation):

•Cold exposure is the physiological way to increase BAT development and its activity. For example, increased BAT has been described for outdoor workers from Northern Europe [75]. However, various questions remain to be answered. At what temperatures BAT is fully activated? How long and often cold exposure needs to be to achieve notable effects on energy expenditure? Van Marken Lichtenbelt [45] for instance reported measurable BAT activity already after mild cold exposure of 16°C for 2 hours and Saito et al. [49] described substantial FDG uptake into BAT after exposure of subjects to 19°C for 2 hours. A significant increase of BAT thermogenesis and energy expenditure has very recently been described after only 3 hours of cold exposure to 18°C [72]. Short term exposure to lower temperatures and chronic cold exposure and is influence on BAT activity will have to be determined in future studies.

•Mimicking cold exposure by pharmacological activation of BAT with sympathomimetics avoids cold exposure of patients probably resulting in higher rates of compliance. β_3_-AR agonists are currently under development and show good results on energy expenditure in rodents. However, clinical trials in humans so far did not provide the expected results, which may be due to different binding characteristics of the human and the rodent β_3_-ARs [76,77]. Selective thyroid hormone mimetics such as GC-1 and KB-141 with reduced activity on heart and muscle promote weight loss in rodents [78,79]. Targeting of the bile acid receptor TGR5 expressed in BAT leads to an increase in energy expenditure via D2 activation. Currently, various ligands on TGR5 are under investigation [73]. Substances that activate the cGMP-signaling pathway (e.g. NPs, PDE5 inhibitors) are further examples of promising substances.

•Activating residing brown fat progenitors in skeletal muscle and WAT is another approach which could be envisioned. Among others, a cytokine in focus is BMP7. BMP7 (OP-1TM) is therapeutically used in the U.S. for the induction of bone growth and osteoporosis treatment. Recently, it has been shown that BMP7 regulates the differentiation of precursor cells into brown adipocytes. *Bmp7*-transgenic mice develop more BAT and have increased energy expenditure and reduced weight gain [57]. Manipulation of COX2-induced PG signaling of BAT progenitors localized in WAT represents an alternative strategy for enhancing BAT activity that could help protecting against energy surplus and body weight gain [61].

•Transdifferentiation of white adipocytes into ‘brite’ adipocytes: Agonists of the transcription factor PPARγ, such as the TZDs rosiglitazone or pioglitazone, increase the expression of brown fat-specific proteins (e.g. PGC1α and UCP1) both in fat cell lines and in white adipose tissue from mice [80,81]. Also a potential to trigger transdifferentiation of white adipocytes towards a brown fat phenotype via the stabilization of the PRDM16 protein has been proposed for TZDs [63]. PPARγ agonists probably increase the metabolic rate by stimulating BAT activity and increase whole body BAT content. However, people with type 2 diabetes treated with pioglitazone at 45 mg/d for 24 weeks showed no changes in their metabolic rate, gained weight, and suffered from fluid retention [82]. The CB1 antagonist rimonabant acts on white adipocytes and leads to a transdifferentiation towards a brown fat phenotype *in vitro*. Patients treated with rimonabant show weight loss and improved glucose homeostasis (RIO study [83,84]) what might be explained by increased thermogenesis, however, central effects are probably also involved. In 2006 the European Medicines Agency (EMA) recommended the approval of rimonabant, the first drug of this class, as an adjunct to diet and exercise in treating obesity. However, in October 2008, EMA recommended the suspension of rimonabant because of its psychiatric side effects [85]. The NPRA has been implicated in the transdifferentiation process of human white adipocytes *in vitro* and in mouse models *in vivo*. Targeting NPRs (or subsequent cGMP signaling) is a promising new approach. Inhibition of PDE5 might by a strategy to interfere with cGMP signaling, however, no human data are available so far.

•Also an *ex vivo* approach seems feasible. Progenitor cells can be isolated from liposuctions or muscle biopsies of an obese donor. These cells can be induced to differentiate *in vitro* into brown adipocytes by treatment with BMP7 or overexpression of *PRDM16* or *PGC1A*. Differentiated brown adipocytes can be transplanted back to the donor where they develop into functional BAT. This approach yielded remarkable results when tested in mice [56].

•Targeting the CNS e.g. the leptin signaling pathways in the hypothalamus might produce SNS effects specific to BAT. In rodents central application of T3 or BMP8B to hypothalamic nuclei resulted in BAT activation by modulation of hypothalamic regulatory circuits [41,42].

**Table 1:** Possible treatments directly targeting WAT and/or BAT in obesity and type 2 diabetes

**Drug**	**Effect**	**Mechanism of action**	**Target organ**	**Class**
BMP7 (OP-1TM)	BAT activation	Developmental regulator	BAT	Cytokine
BMP8B	BAT activation	Developmental regulator	BAT, CNS	
Sildenafil, vardenafil	WAT (trans-) differentiation, (BAT activation)	PDE5 inhibition	WAT, (BAT)	Enzyme inhibitor
Amrinone	WAT lipolysis, (WAT transdifferentiation, BAT activation)	PDE3 inhibition	WAT, (BAT)	
GC-1, KB-141	BAT activation, (WAT transdifferentiation)	Thyroid hormone mimetics	BAT	Hormone
T3	BAT activation, (WAT transdifferentiation)	Thyroid hormone	BAT, CNS	
BRL-26830, L-796568, N-5984	BAT activation, WAT transdifferentiation, lipolysis	selective β_3_-AR agonist	BAT, WAT	Receptor agonist
Bile acids (endogenous ligands)	BAT activation	TGR5 receptor agonist	BAT	
ANP, BNP (endogenous ligands)	BAT activation, WAT transdifferentiation	NPRA agonsit	BAT, WAT	
Rimonabant	WAT transdifferentiation	CB1 antagonist	WAT, CNS	Receptor antagonist
TZDs, NS-1, balaglitazone, MRL-24, (Sulfonylureas)	WAT (trans-) differentiation, BAT activation	(partial) activation of PPARγ	WAT, BAT	Transcription factor ligand

## Conclusions

Direct targeting of adipose tissue is still far from being a magic bullet for the treatment of obesity and type 2 diabetes. However, new options arise by targeting BAT to increase energy expenditure. Nevertheless, several questions remain to be answered before targeting of BAT as therapeutic option becomes possible. It is still not clear if chronic BAT activation leads to sufficient energy expenditure to achieve the therapeutic goal of weight loss. Furthermore, it is not known if compensatory mechanisms such as increased appetite might countervail increased energy expenditure. Well-directed cold exposure appears to be the most physiological stimulus to activate BAT. Optimum conditions for a targeted cold exposure of an anticipated “cold-therapy” need to be further elucidated. A pharmacological and cell-based therapy approach seems plausible and many druggable targets could be identified so far. On the other hand, many safety concerns remain, requiring the need for the identification of new drug targets and substances in line with a careful benefit-risk assessment.

## Abbreviations

AC: adenylate cyclase; AKT1: protein kinase B; AMPK: AMP-activated protein kinase; ATP: adenosine triphosphate; BA: bile acids; BAT: brown adipose tissue; BMI: body mass index; BMP: bone morphogenic protein; C/EBPβ: CCAAT/enhancer binding protein β; cAMP: cyclic adenosine monophosphate; CB1: cannabinoid type 1 receptor; CDK5: cyclin-dependent kinase 5; cGMP: cyclic guanosine monophosphate; CNS: central nervous system; COX2: cyclooxygenase 2; CT: computer tomography; D2: deiodinase type 2; EMA: European Medicines Agency; FAS: fatty acid synthase; FDG: 2-deoxy-2[^18^F]fluoro-D-glucose; FFA: free fatty acids; Fndc5: fibronectin type III domain containing 5; GC: guanylate cyclase; GLUT: glucose transporter; HBA1c: hemoglobin A1c; HSL: hormone-sensitive lipase; IL: interleukin; iNOS: inducible NO synthase; IR: insulin receptor; IRS: insulin receptor substrate; MAPK: mitogen-activated protein kinase; MSCs: mesenchymal stem cells; NE: norepinephrin; NEFA: nonesterified fatty acid; NO: nitric oxide; NPRA: NP receptor A; NPRC: NP clearance receptor; NPs: natriuretic peptides; p38MAPK: p38 mitogen-activated protein kinase; PDE: phosphodiesterase; PET: positron emission tomography; PGC1α: PPARγ coactivator 1α; PGs: prostaglandines; PI3K: phospoinositide 3 kinase; PKA: protein kinase A; PKCζ: protein kinase C ζ; PKG: protein kinase G; PPARγ: peroxisome proliferator-activated receptor γ; PRDM16: PR domain containing 16; PTP1B: protein tyrosin phosphatase 1B; RhoA: ras homolog gene family member A; ROCK: rho-associated protein kinase; SNS: sympathetic nervous system; T3: triiodothyronine; T4: thyroxine; TG: triacylglycerol; TGR5: G-protein-coupled receptor 5; TNFα: tumor necrosis factor α; TR: thyroid hormone receptor; TZDs: thiazolidinediones; UCP1: uncoupling protein 1; WAT: white adipose tissue; β-AR: β-adrenoceptor.

## Competing interests

The authors declare no financial conflict of interest.

## Authors’ contributions

BH wrote the manuscript and prepared figures, PS reviewed the manuscript and prepared figures, PM reviewed the manuscript, NE reviewed the manuscript. All authors read and approved the final manuscript.
